# Supramolecular Control over the Interparticle Distance in Gold Nanoparticle Arrays by Cyclodextrin Polyrotaxanes

**DOI:** 10.3390/nano8030168

**Published:** 2018-03-16

**Authors:** Joao Paulo Coelho, José Osío Barcina, Elena Junquera, Emilio Aicart, Gloria Tardajos, Sergio Gómez-Graña, Pablo Cruz-Gil, Cástor Salgado, Pablo Díaz-Núñez, Ovidio Peña-Rodríguez, Andrés Guerrero-Martínez

**Affiliations:** 1 Departamento de Química Física, Universidad Complutense de Madrid, Avenida Complutense s/n, 28040 Madrid, Spain; joaopc@uni-muenster.de (J.P.C.); junquera@quim.ucm.es (E.J.); aicart@quim.ucm.es (E.A.); tardajos@quim.ucm.es (G.T.); 2 Departamento de Química Orgánica, Universidad Complutense de Madrid, Avenida Complutense s/n, 28040 Madrid, Spain; josio@quim.ucm.es (J.O.B.); pablocru@estumail.ucm.es (P.C.-G.); castorsalgado@ictp.csic.es (C.S.); 3 Departamento de Química en Ciencias Farmacéuticas, Universidad Complutense de Madrid, Plaza Ramón y Cajal s/n, 28040 Madrid, Spain; segome02@ucm.es; 4 Instituto de Fusión Nuclear, Universidad Politécnica de Madrid, José Gutiérrez Abascal 2, 28006 Madrid, Spain; p.diazn@alumnos.upm.es (P.D.-N.); ovidio.pena@upm.es (O.P.-R.)

**Keywords:** supramolecular chemistry, self-assembly, gold nanoparticle, cyclodextrin, polyrotaxane, array, hot spot, surface-enhanced spectroscopy

## Abstract

Amphiphilic nonionic ligands, synthesized with a fixed hydrophobic moiety formed by a thiolated alkyl chain and an aromatic ring, and with a hydrophilic tail composed of a variable number of oxyethylene units, were used to functionalize spherical gold nanoparticles (AuNPs) in water. Steady-state and time-resolved fluorescence measurements of the AuNPs in the presence of α-cyclodextrin (α-CD) revealed the formation of supramolecular complexes between the ligand and macrocycle at the surface of the nanocrystals. The addition of α-CD induced the formation of inclusion complexes with a high apparent binding constant that decreased with the increasing oxyethylene chain length. The formation of polyrotaxanes at the surface of AuNPs, in which many α-CDs are trapped as hosts on the long and linear ligands, was demonstrated by the formation of large and homogeneous arrays of self-assembled AuNPs with hexagonal close packing, where the interparticle distance increased with the length of the oxyethylene chain. The estimated number of α-CDs per polyrotaxane suggests a high rigidization of the ligand upon complexation, allowing for nearly perfect control of the interparticle distance in the arrays. This degree of supramolecular control was extended to arrays formed by AuNPs stabilized with polyethylene glycol and even to binary arrays. Electromagnetic simulations showed that the enhancement and distribution of the electric field can be finely controlled in these plasmonic arrays.

## 1. Introduction

In the last decades, gold nanoparticles (AuNPs) have been actively investigated due to their interesting optical properties and promising technological applications [1]. Such optical features are dominated by the so-called localized surface plasmon resonance (LSPR), i.e., the collective oscillation of conduction electrons in resonance with the incoming light [2]. The wavelength of the LSPR band is very sensitive to several parameters, such as the size, shape, and surrounding medium of the AuNPs [3]. Remarkably, the excitation of the LSPR is accompanied by the confinement of light and a large enhancement of the electric field near the AuNP boundary [4]. Compared to isolated AuNPs, their assemblies provide a much higher enhancement due to the coupling between the LSPRs of adjacent particles, resulting in distance-dependent large electromagnetic fields, the so-called hot spots [5]. In this context, two-dimensional (2D) ordered AuNP arrays provide an excellent platform for the development of plasmonically active substrates with intense hot spots [6], which find many applications in areas such as surface-enhanced spectroscopy techniques [7,8]. The crystallographic parameters of the assembly, including the nanocrystal size and periodicity and the interparticle distance, are critical determinants for the final performance of the array [9]. However, the control of large and ordered areas of assembled AuNPs with specific interparticle distances remains a challenging task [10,11]. Such a lack of control has a detrimental effect on the spectral features and wavelength of the LSPR band of the ensemble relative to that of an ideal array [12]. Therefore, investigations in this regard may reinforce the feasibility of applications of AuNP arrays, which usually demand the optically controlled coupling of the LSPR band wavelength and that of the light excitation source [13].

Self-assembly is recognized as one of the most general strategies toward the generation of ordered nanoparticle arrays [14], where the driving force of the process is the minimization of the free energy of the final assembled structure [15]. Specifically, the so-called directed self-assembly refers to the rational selection of particle building blocks to obtain a particular nanostructure [16] by exploiting characteristic properties encoded in the nanocrystals themselves, such as the size and shape [17] or the surface chemistry [18]. Most strategies for the directed self-assembly of AuNPs are based on the rational functionalization of the nanocrystal surface with a library of organic molecules [19], polymers [20], and biomolecules [21]. Under appropriate physicochemical conditions (i.e., solvent [22], temperature [23], concentration [24], pH [25], etc.), such ligands contribute to the self-assembly of particles via specific and directional interactions, i.e., short range (van der Waals [22], coulomb [16], hydrogen bonding [23,25], and/or hydrophobic interactions [17,24]) and long range (capillarity [26] or flow dragging [27]). Through these interactions, AuNPs have shown remarkable versatility for functionalization with supramolecules [28], allowing for the directed self-assembly of nanocrystals into a variety of complex superstructures, such as plasmonic polymers [6], membranes [29], arrays [30], and supercrystals [31].

**Scheme 1:**
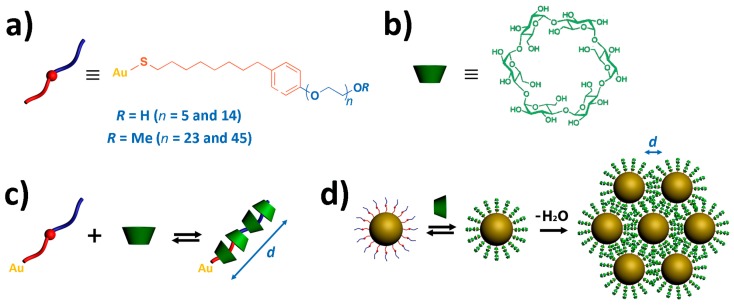
(**a**) Chemical structure of the nonionic ligands Ige*_n_*S-linked to gold nanoparticles (AuNPs) where the hydrophobic and hydrophilic regions are shown in red and blue, respectively (*n* represents the number of oxyethylene units in the hydrophilic chain; the substituent *R* is a hydrogen atom for *n* = 5 and 14 and a methyl group for *n* = 23 and 45); (**b**) Chemical structure of α-cyclodextrin (α-CD) formed by six glucose units; (**c**) Structure of the polyrotaxane obtained by complexation of Ige*_n_*S-, linked to AuNPs, and α-CD, where *d* represents the length of the fully extended supramolecule; (**d**) Schematic preparation of an array of polyrotaxane-stabilized AuNPs where *d* represents the interparticle distance of the hexagonal packing between the surfaces of AuNPs.

Among the different molecular entities used to prepare supramolecules, cyclodextrins (CDs) stand out because of their excellent ability to form inclusion complexes with a variety of guest molecules in aqueous solution [32,33,34]. These cyclic oligosaccharides are built up from α-d-glucopyranose residues linked by glycosidic bonds, with the most common being those formed by six, seven, or eight glucose units (α-, β-, and γ-CD, respectively) [35]. Structurally, CDs display a hollow truncated cone shape with a hydrophobic cavity and two hydrophilic rims (Scheme 1). Thus, CDs constitute a singular microenvironment where molecules with suitable size and hydrophobic character can be hosted [36,37]. As relevant supramolecular examples, CDs have been widely used as hosts trapped on long and linear molecules, i.e., molecular axes without bulky end-groups, forming polyrotaxanes [38]. In these complexes, not only do host–guest interactions add to the overall stability of polyrotaxanes but so does the cooperative forces resulting from the formation of a hydrogen bond network between adjacent CDs [39]. Interestingly, this phenomenon decreases the solubility of the complexes by reducing the hydration states and increasing the molecular rigidity, usually leading to the directed self-assembly of polyrotaxanes into solids [40]. Therefore, CDs and their rotaxanes are ideal candidates for the preparation of supramolecular nanoarchitectures with structural and functional properties [41].

Along these lines, we have recently reported a supramolecular chemistry-based strategy to prepare highly ordered arrays of spherical AuNPs stabilized by short polyrotaxanes [31]. These supramolecules are formed at the surface of the particles by a directly linked nonionic ligand entrapping several α-CDs (Scheme 1) [42]. Under evaporation of this colloid, the AuNPs self-assemble into a hexagonal 2D array where the interparticle distance matches the length of the ligand when completely extended and threaded by the macrocycles. Such self-assembly is driven by weak intermolecular interactions between polyrotaxanes of adjacent AuNPs due to the spatial anisotropy of the dielectric properties in CD polyrotaxanes [43]. In contrast, the interparticle gap was found to be clearly out of control in the absence of α-CD, leading to a loss of the array periodicity. In this respect, we sought to elucidate whether the length of the polyrotaxane, which is directly related to the length of the nonionic ligand and the number of threaded macrocycles, could be used to control the interparticle distance in AuNP arrays.

For this purpose, we synthesized a family of nonionic ligands with different lengths by tuning the number of oxyethylene units in the hydrophilic chain, here used to stabilize the spherical AuNPs in water (Scheme 1). The addition of α-CD to such colloids enabled the formation of supramolecular complexes with different and controlled lengths at the surface of the AuNPs. Under solvent evaporation, we observed the formation of highly ordered 2D arrays of AuNPs with hexagonal packing, whose interparticle distance was roughly the length of the corresponding supramolecule. This degree of control was observed even for distances longer than the diameter of the nanocrystals forming the ensemble. This extreme control over the interparticle distance is ideal for controlling the near-field intensity within the gaps. Additionally, to develop a more general methodology, we have used a commercially available polyethylene glycol polymer as the capping agent to form α-CD polyrotaxanes [39] at the AuNP surface. At different α-CD molar ratios, remarkable control over the interparticle distance of the arrays was achieved. Finally, ordered binary arrays were obtained by combining polyrotaxane-stabilized AuNPs with two different nanocrystal sizes, where the small AuNPs were mainly located at the interstitial octahedral holes formed by the hexagonal packing of the larger particles. This type of ensemble provides yet an additional way of controlling the near-field intensity at the interparticle gaps.

## 2. Results and Discussion

### 2.1. Synthesis and Functionalization of Gold Nanoparticles

We synthesized a family of dimeric nonionic precursors (Ige*_n_*S)_2_ (see Materials and Methods section) derived from the structure of the commercially available Igepal surfactant [40,42] (Scheme 1). The disulfide functionalization ensures, after scission in the presence of AuNPs, the strong coordination of the monomeric ligand Ige*_n_*S- to gold [44], maintaining the hydrophobic moiety comprising the alkyl chain and aromatic ring close to the AuNP surface. On the opposite end of the molecule, the hydrophilic chain with different numbers of oxyethylene units (*n* = 14, 23, and 45) introduces control over the length of the ligand. For simplicity of the synthetic protocol, in the case of the larger ligand (Ige_45_S-), the oxyethylene chain bears a terminal methyl group instead of the typical hydroxyl functionalization of their shorter counterparts (Scheme 1). Due to the non-bulky character of the ending methyl and hydroxyl groups, we would not expect significant differences in the inclusion process of both ligands with α-CD [36] (Scheme 1).

**Figure 1:**
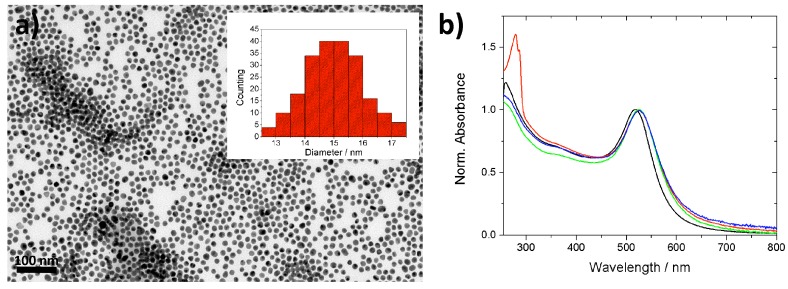
(**a**) Representative TEM micrograph and size distribution (inset) of citrate-stabilized gold nanoparticles (AuNPs) (15 ± 2 nm in diameter); (**b**) Normalized UV-vis absorption spectra of spherical AuNPs (1 nM) before (red) and after (black) functionalization with Ige_5_S- (3 mM), after removal of the excess Ige_5_S- (surface concentration ~1 µM, green), and after subsequent addition of α-cyclodextrin (α-CD) (1 mM, blue).

The synthesized Ige*_n_*S-ligands and the previously reported ligand Ige*_5_*SH [31] were used to coat monodisperse spherical AuNPs (Ige*_n_*S-AuNPs) of 15 ± 2 nm in diameter, previously synthesized under citrate-based conditions (Figure 1a; see Materials and Methods section) [45]. Figure 1b shows the UV-vis absorption spectrum of the Ige_5_S-AuNP colloid (3 mM of Ige_5_S- and 1 nM of AuNPs), where the two bands at 280 and 524 nm correspond to the characteristic absorption of the ligand and the AuNP LSPR, respectively. Upon functionalization, the LSPR band is red-shifted by 7 nm compared to the citrate-synthesized AuNPs (Figure 1b), which is consistent with an increase in the local refractive index of the AuNPs after molecular replacement [46]. Interestingly, no significant changes in the LSPR band were observed after removal of the excess Ige_5_S- (final surface concentration of the ligand ~1 µM) and subsequent addition of α-CD (even at high concentrations such as 1 mM) (Figure 1b), which renders UV-vis spectroscopy an unsuitable technique for the investigation of the formation of supramolecular complexes at the AuNP surface (CD-Ige*_n_*S-AuNPs). Analogous optical features for the different Ige*_n_*S-AuNP colloidal solutions in the absence and presence of α-CD were observed independent of the length of the nonionic ligand (Table 1).

### 2.2. Cyclodextrin Supramolecular Complexation at the Gold Nanoparticle Surface

**Figure 2:**
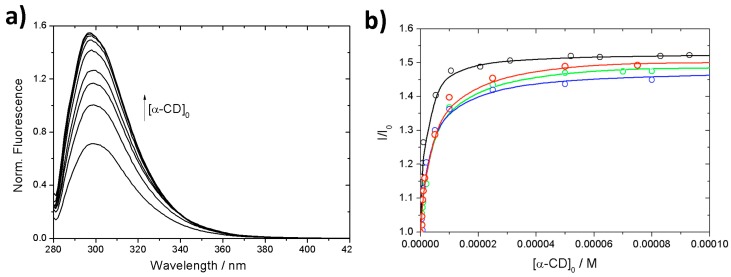
(**a**) Emission spectra of CD-Ige_5_S-AuNPs at different concentrations of α-CD (~1 µM of Ige_5_S-, 1 nM of AuNPs, and 1–100 µM of α-CD; *λ*_exc_ = 280 nm); (**b**) Intensity ratios at 300 nm of CD-Ige*_n_*S-AuNPs (*n* = 5 in black, *n* = 14 in red, *n* = 23 in green, and *n* = 45 in blue) versus the total concentration of α-CD. The solid lines show the corresponding fits to Equation (1).

Fluorescence measurements can provide more detailed information about the complexation process at the AuNP surface. In the absence of α-CD, the steady-state fluorescence spectrum of Ige_5_S-AuNPs presents a wide band with a maximum at 300 nm (Figure 2a) [31]. The fluorescence emission of the system in the presence of α-CD was studied by maintaining constant the concentration of Ige_5_S-AuNPs (~1 µM of Ige_5_S- and 1 nM of AuNPs). When variable amounts of the macrocycle were added, an increase in the fluorescence intensity was observed (Figure 2a), indicating the formation of complexes with higher quantum yields, attributed to the inclusion of the aromatic group in the hydrophobic cavity of α-CD [40]. This suggests that the macrocycles are initially trapped by the oxyethylene chain but then move towards the hydrophobic region of the ligand (Scheme 1). The fluorescence intensity ratio (*I*/*I*_0_) at 300 nm is plotted in Figure 2b versus the macrocycle concentration. Considering the reported polyrotaxane structure formed by conventional Igepal surfactants and a number of adjacent β-CDs [42] in which the benzene group interacted directly with only one macrocycle, an apparent 1:1 stoichiometry between the aromatic group and α-CD can be assumed, in which both the free and complexed forms of the aromatic moiety are fluorescent. Therefore, the change registered in the fluorescence intensity is given by

(1) $$\frac{I}{I_{0}}=\frac{1+aK_{b}\left[\mathrm{CD}\right]}{1+K_{b}\left[\mathrm{CD}\right]}$$

with *I* is the measured fluorescence of CD-Ige*_n_*S-AuNPs at each α-CD concentration, *I*_0_ is the fluorescence of Ige*_n_*S-AuNPs, [CD] is the concentration of the macrocycle not interacting with the aromatic group (in the free form or complexed form at the aliphatic and/or oxyethylene regions), and *K*_b_ is the apparent association constant between the aromatic group and one α-CD. The parameter *a* is a function of the quantum yield (*ϕ_i_*) and absorptivity (*ε_i_*) of CD-Ige*_n_*S-AuNPs and Ige*_n_*S-AuNPs through *a* = *ε*_CD-Ige_*_n_*_S-AuNP_ *ϕ*_CD-Ige_*_n_*_S-AuNP_ */ε*_Ige_*_n_*_S-AuNP_ *ϕ*_Ige_*_n_*_S-AuNP_.

The general mass balance and mass action law of a 1:1 equilibrium link the concentrations of all the components in solution, and the apparent binding constant can be estimated directly by nonlinear fitting of the fluorescence intensity variation versus the total concentration of the macrocycle, [CD]_0_ [47]. In the case of the fluorescence of CD-Ige_5_S-AuNPs, the fitted curve is shown in Figure 2b, and the resulting estimated parameters are *K*_b_ = (3.7 ± 0.4) × 10^4^ L mol^−1^ and *a* = 1.52 ± 0.01. Such a high value for the apparent binding constant is in good agreement with literature data obtained for conventional nonionic surfactants and CDs [48], confirming the easy penetration of the oxyethylene chain through α-CD in the case of short ligands. The fitted parameter *a* > 1 confirms the increase in the quantum yield upon formation of the complex as a consequence of the dehydration of the aromatic moiety, considering that the absorptivity of the free and complexed forms may be similar [40].

The complexation process was also investigated by fluorescence lifetime measurements of the above-mentioned colloids (see Materials and Methods section). The fluorescence decay curves of CD-Ige_5_S-AuNPs in the absence and excess of α-CD (1.0 mM) were fitted with a single exponential displaying decay times of 4.0 and 6.4 nm, respectively (Table 1). Such a lifetime increase may be related to a new emissive species with high quantum yield corresponding to an inclusion complex in excess of the macrocycle, and is in good agreement with the fitted parameter *a* > 1 obtained from Figure 2b.

Analogous steady-state and time-resolved fluorescence experiments were performed for the AuNPs stabilized with the longer ligands (Table 1). In all cases, upon addition of α-CD, the intensity of the emission spectra of the corresponding Ige*_n_*S-AuNPs increased until a plateau was reached (Figure 2b), suggesting the complete complexation of the aromatic group. From the fitted curves according to Equation (1), a reduction in the apparent binding constant between the aromatic moiety and α-CD is observed relative to the shortest ligand (Table 1). This suggests a hindrance of the macrocycle mobility on the molecular axis attached to the AuNP with the increasing number of oxyethylene units. In contrast, the parameter *a* remains nearly constant independently of the length of the oxyethylene chain, showing analogous increments in the quantum yields (Table 1) and, therefore, similar microenvironments for the aromatic group upon complexation. This is also confirmed by similar increases in the lifetimes of all the Ige*_n_*S-AuNP samples (Table 1).

### 2.3. Polyrotaxane-Mediated Self-Assembly of Gold Nanoparticles into 2D Arrays

**Figure 3:**
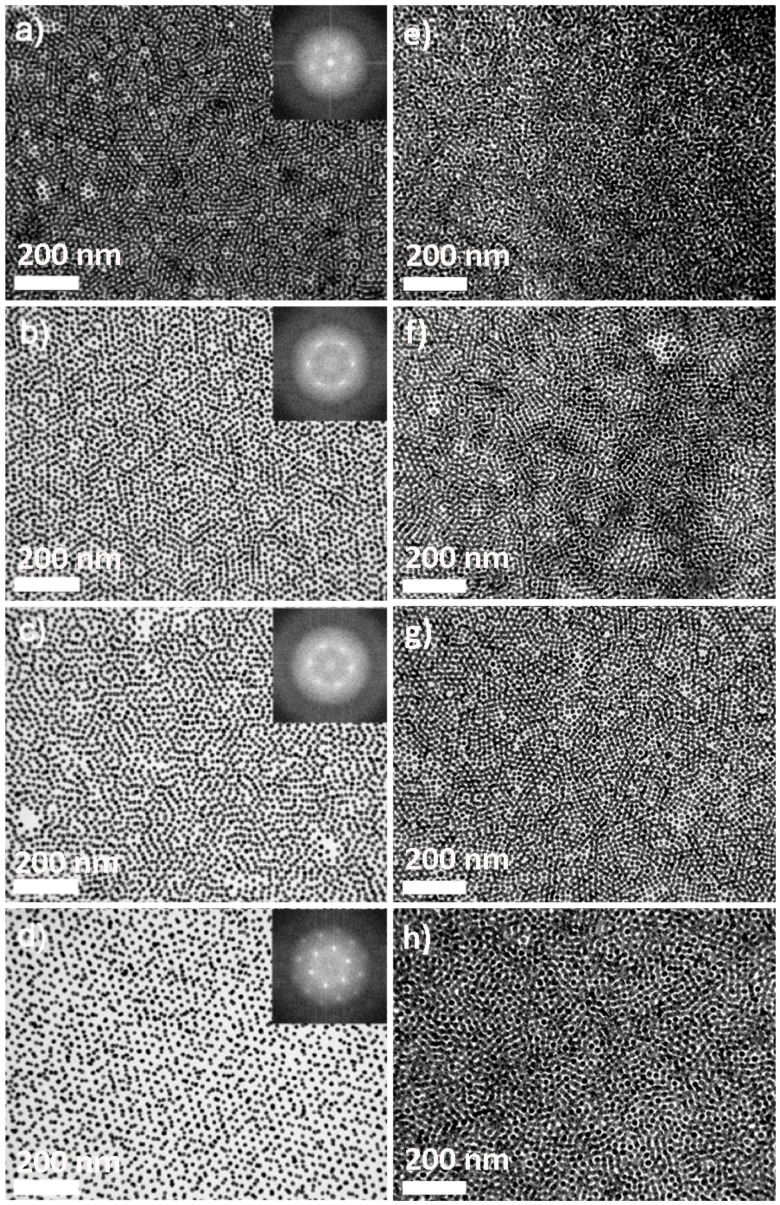
Representative TEM micrographs of CD-Ige*_n_*S-AuNP arrays (~1 µM of Ige*_n_*S-, 1 nM of AuNPs, and 1 mM α-CD) for the ligands with (**a**) 5; (**b**) 14; (**c**) 23; and (**d**) 45 oxyethylene units. The insets show the corresponding fast Fourier transform (FFT) of the images. TEM micrographs of Ige*_n_*S-AuNP arrays for the ligands with (**e**) 5; (**f**) 14; (**g**) 23; and (**h**) 45 oxyethylene units.

Once demonstrated that complexes between Ige*_n_*S- ligands and α-CD are formed at the surface of AuNPs, the presence of polyrotaxanes at high stoichiometries was evaluated by simple drop-casting of the corresponding CD-Ige*_n_*S-AuNP colloids (~1 µM of Ige*_n_*S-, 1 nM of AuNPs, and 1 mM α-CD) on carbon-coated TEM grids (Figure 3). By simple drop-casting on carbon-coated TEM, the spontaneous formation of self-assembled layers of CD-Ige*_n_*S-AuNPs was observed independently of the stabilizing ligand. While short-range ordered assemblies of AuNPs with interparticle distances of ca. 2–3 nm were detected for all the ligands in the absence of the macrocycle, well-defined and ordered arrays of AuNPs were formed in the presence of α-CD. This clearly shows the high flexibility of the free oxyethylene chains, which bend around the AuNPs during the colloidal evaporation process.

Figure 3 shows representative TEM micrographs of highly ordered arrays of CD-Ige*_n_*S-AuNPs with hexagonal packing. Noticeably, the interparticle distance of the 2D arrays increases with the length of the corresponding Ige*_n_*S-ligand (Table 2). The fast Fourier transform (FFT) of the images confirms a nearly perfect hexagonal geometry with an average distance between the centers of neighboring CD-Ige*_n_*S-AuNPs of 4.1, 7.8, 10.9, and 20.0 (±0.5) nm for the ligands with 5, 14, 23, and 45 oxyethylene units, respectively. Such differences observed in the absence and presence of α-CD indicate the high rigidization of the ligand upon complexation, even in the case of the longest ligand, suggesting the presence of polyrotaxanes with maximum stoichiometry.

**Figure 4:**
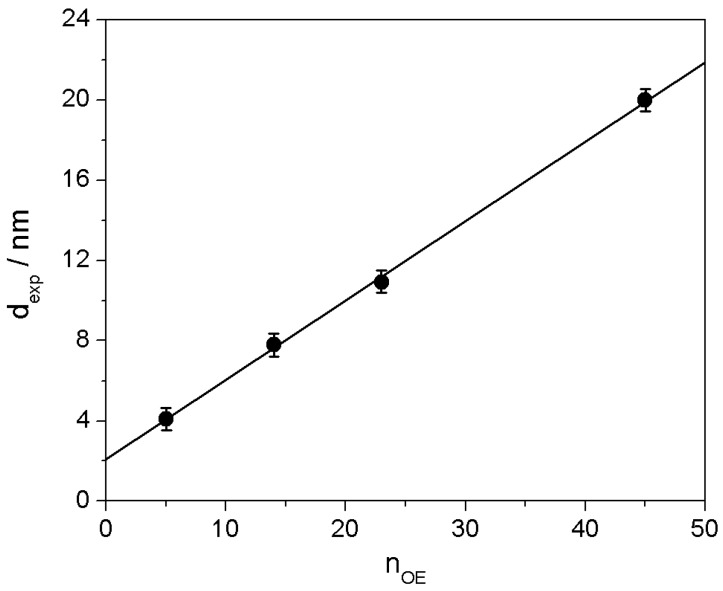
Linear correlation between the interparticle distance and the number of oxyethylene units in the CD-Ige*_n_*S-AuNP arrays. The error bars have been estimated from the FFT analysis of the TEM micrographs.

Taking into account that the alkyl chain (eight methyl groups) and the aromatic ring may thread one α-CD each [42] and that it has been reported that approximately two oxyethylene segments are complexed by one macrocycle [49], we estimated the number of macrocycles that form each polyrotaxane (Table 1). Then, the resulting CD:Ige*_n_*S-AuNP stoichiometries are 5:1, 9:1, 14:1, and 25:1 for the ligands with 5, 14, 23, and 45 oxyethylene units, respectively. From these stoichiometries and considering that the height of α-CD is 0.79 nm [35], we estimated the theoretical interparticle distances of the different CD-Ige*_n_*S-AuNPs arrays (Table 2), which are in perfect agreement with the experimental distances obtained by the FFT analysis of the TEM micrographs. Such consistency was confirmed by plotting the experimental interparticle distance (*d*_exp_) of the different arrays with the number of oxyethylene units of the ligand (*n*_OE_), which showed a linear correlation (*d*_exp_ = 2.09 + 0.39*n*_OE_; *R*^2^ = 0.998) (Figure 4). This fit indicates that the common hydrophobic region in Ige*_n_*S-AuNPs, formed by the aliphatic tail and aromatic group (Scheme 1), threads, as previously considered, approximately two macrocycles (*n*_OE_ = 0, *d*_exp_ = 2.09 nm, *n*_α-CD_ = 2.6).

**Figure 5:**
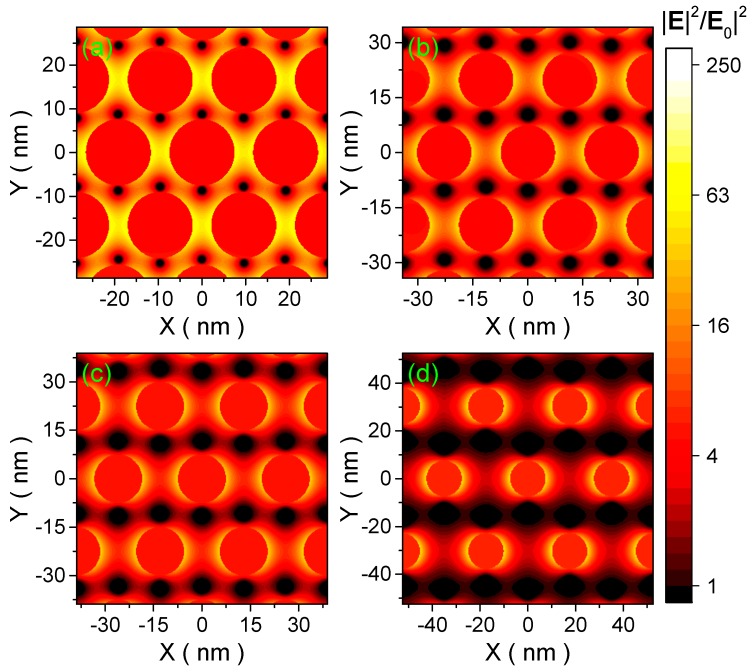
Local field enhancement contour plots in the middle plane for hexagonal arrays of 15-nm AuNPs with interparticle distances of (**a**) 4.2; (**b**) 7.8; (**c**) 10.9; and (**d**) 20.0 nm. The electromagnetic wave is polarized along the *x*-axis and impinges along the *z*-axis.

Figure 5 depicts the near-field enhancement of the electric field for hexagonal arrays of AuNPs (diameter of 15 nm) with various interparticle distances corresponding to those obtained experimentally: 4.1, 7.8, 10.9, and 20.0 nm (see Materials and Methods section). These results were calculated using the Multiple Sphere T-Matrix (MSTM) 3.0 computer code based on the T-matrix formalism [50,51]. The enhancement of the near-field in the interparticle gaps reaches values of around 200, 100, 80, and 60 for separations of 4.1, 7.8, 10.9, and 20.0 nm, respectively, as observed in the near-field maps. Hence, it is clear that this methodology allows the extremely precise variation of the electric field intensity within a wide range, which is very appealing for applications in surface-enhanced spectroscopy methodologies [7,8].

### 2.4. 2D Arrays Based on the Cyclodextrin Complexation of Polyoxyethylene Glycol-Functionalized Gold Nanoparticles

**Figure 6:**
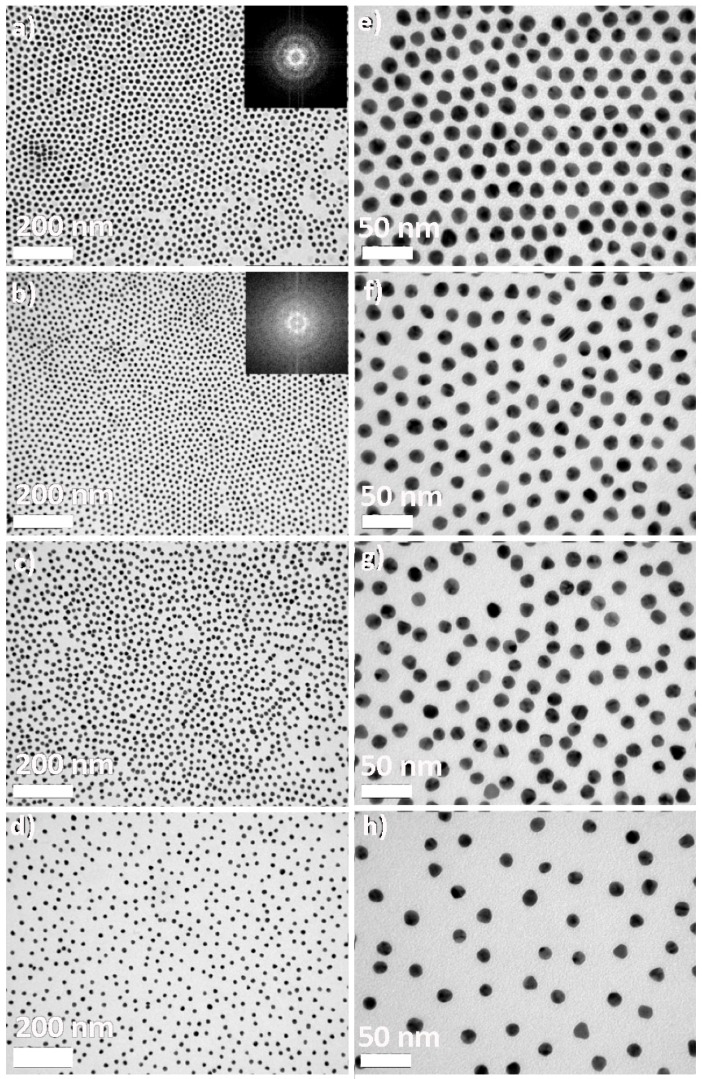
Representative TEM micrographs of CD-PEG*_n_*S-AuNP arrays (~1 µM of PEG_114_SH and 1 nM of AuNPs) in the presence of (**a**,**e**) 5 µM; (**b**,**f**) 50 µM; (**c**,**g**) 0.5 mM; and (**d**,**h**) 5 mM of α-CD. The insets show the corresponding FFT of the images. No hexagonal patterns were observed above 50 µM of α-CD.

It is well known that polyoxyethylene glycol polymers form highly stable polyrotaxanes with α-CD in aqueous solution [39]. Therefore, we wondered whether it would be possible to easily control the interparticle distance of such AuNP arrays with this type of supramolecule by finding a balance between complexed and non-complexed regions, which may be potentially achieved at different macrocycle concentrations. For this aim, we coated the previously investigated AuNPs with a commercially available polyoxyethylene glycol polymer with 114 oxyethylene units functionalized with a thiol group (CD-PEG_114_S-AuNPs). In good agreement with the previous results, drop-casting of the CD-PEG_114_S-AuNP colloid at different concentrations of α-CD (~1 µM of PEG_114_SH, 1 nM of AuNPs, and 5 µM–5 mM of α-CD) typically resulted in the formation of hexagonal 2D arrays where the interparticle distance increased with the concentration of the macrocycle (Figure 6). Analogously to the CD-Ige*_n_*S-AuNP system, a short-range ordered assembly of AuNPs was formed in the absence of the macrocycle under the same experimental conditions (not shown). The FFT analysis of the TEM micrographs revealed successful crystallinity of the AuNP assembly up to a concentration of ca. 0.1 mM of α-CD (with interparticle distances of 6.7 ± 0.5 nm and 10.6 ± 0.5 nm for 5 and 50 µM of α-CD, respectively). Above such concentration, the deposited AuNPs lose the crystal periodicity, most likely due to the high length and flexibility of the selected polymeric ligand, although the homogeneous dispersion of AuNPs is maintained (Figure 6).

**Figure 7:**
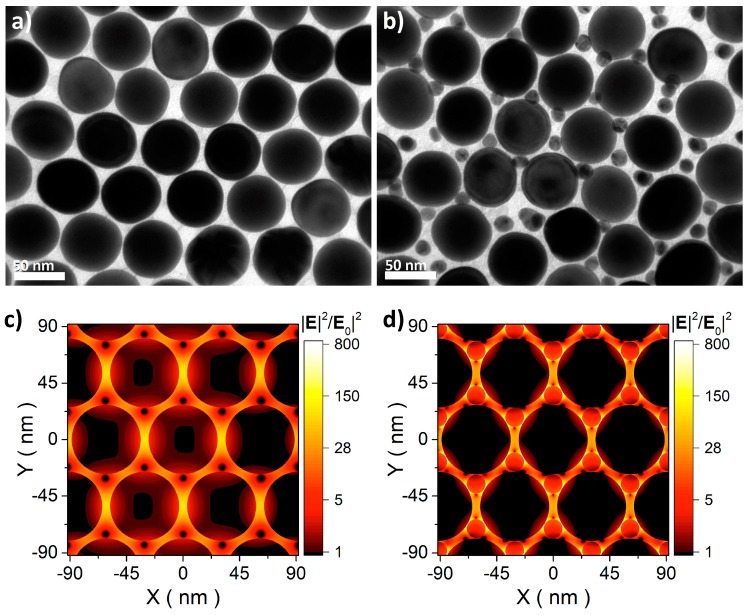
Representative TEM micrographs of arrays of (**a**) CD-PEG_114_S-AuNPs (55 ± 5 nm), and (**b**) CD-PEG_114_S-AuNPs (55 ± 5 nm and 15 ± 2 nm at a 1:1 ratio). Local field enhancement contour plots in the middle plane for a (**c**) hexagonal array of 55 nm AuNPs with an interparticle distance of 6 nm and (**d**) after inclusion of 15 nm AuNPs. The electromagnetic wave is polarized along the *x*-axis and impinges along the *z*-axis.

Spherical AuNPs with larger sizes (>50 nm) concentrate higher electromagnetic fields near the particle boundary and, therefore, they produce very intense hot spots when assembled at short distances [4]. Thus, the preparation of 2D arrays with these AuNPs is particularly appealing for surface-enhanced spectroscopy applications [7,8]. In this context, a 2D array of spherical AuNPs typically shows hexagonally close packing with a minimum interparticle distance, where the occupied fraction is ~74% (Figure 7a). Hence, ~26% of the unit cell volume within the ensemble is unoccupied, which consists of octahedral (~17%) and tetrahedral (~9%) holes. Considering that the radius of octahedral holes is ca. 0.41 times that of AuNPs, the inclusion of small nanocrystals with commensurate sizes to the dimension of the octahedral holes may enhance the packing fraction of the array up to ~90%, and subsequently the number and intensity of formed hot spots [52].

In this context, and considering that long polyoxyethylene glycol polymers functionalized with a thiol group have been extensively employed to coat AuNPs with large sizes [53], we envisioned the combination of two size distributions of CD-PEG_114_S-AuNPs with suitable dimensions (55 ± 5 nm and 15 ± 2 nm in diameter) to fully occupy the 2D array space (see Materials and Methods section). Firstly, the spontaneous formation of self-assembled layers of large CD-PEG_114_S-AuNPs was successfully tested under the previously optimized experimental conditions (~1 µM of PEG_114_SH, 1 nM of AuNPs, and 5 µM of α-CD). Figure 7a shows representative TEM micrographs of highly ordered arrays of large CD-PEG_114_S-AuNPs with hexagonal packing where octahedral holes with a diameter of ca. 22 nm can be perfectly distinguished. The TEM micrograph shows an interparticle distance of 6.2 ± 0.5 nm, in good agreement with that previously obtained for small CD-PEG_114_S-AuNPs under analogous experimental conditions. Upon mixing both types of CD-PEG_114_S-AuNPs at a 1:1 ratio (1 nM of AuNPs), a well-defined and ordered binary array of AuNPs is obtained (Figure 7b), in which the smaller nanocrystals are mainly located at the octahedral holes of the ensemble, enhancing the packing fraction of the array. Interestingly, clear segregation of the AuNPs by size was observed in the absence of the macrocycle (not shown), which reinforces the convenience of using cyclodextrin polyrotaxanes for supramolecular control of the AuNP interparticle distance.

Simulations of the near-field enhancement for the homogeneous array and that containing smaller particles are depicted in Figure 7c,d, respectively. Two important advantages are gained in the latter case. Firstly, the calculated enhancement of the electric field is approximately two-fold in between the large and small AuNPs, whereas it is barely affected in the gap of large AuNPs. Secondly, the number of hot spots has considerably increased (three new hot spots per small nanocrystal). Hence, with the small inclusions, a larger and more homogeneous electric field is obtained, which implies that this system should behave considerably better than one without inclusions for any given surface-enhanced technique.

In summary, we have shown that the formation of α-CD polyrotaxanes at the surface of AuNPs stabilized with oxyethylene nonionic ligands is a powerful tool to control the interparticle distance in AuNP arrays at the molecular level. The rigidization of the supramolecules by the formation of a network of adjacent complexed α-CDs was found to be the key parameter for the self-assembly of AuNPs into hexagonal closely packed arrays. This effect was demonstrated using AuNPs stabilized by ligands with different numbers of oxyethylene units. We were thus able to control interparticle distances that were even larger than the diameter of the AuNPs forming the ensemble. This degree of supramolecular control was also extended to large AuNPs and binary systems made of differently sized AuNP populations. This extreme structural control translates into fine control over the intensity and distribution of the electric field, which is of paramount importance in surface-enhanced spectroscopies. These techniques, in turn, have ample applications in fields such as sensing and photonics, to name only a couple. By selecting the appropriate dimension, morphology, and supramolecular functionalization of the AuNPs, we anticipate the use of these plasmonic arrays as powerful substrates for surface-enhanced Raman scattering spectroscopy upon attachment to biomolecules, such as proteins or DNA [54,55], for structure determination.

## 3. Materials and Methods

### 3.1. Characterization Techniques

- ^1^H and ^13^C NMR measurements: ^1^H and ^13^C NMR spectra (see Supplementary Materials) were recorded on a Bruker Avance DPX-300 spectrometer (300 MHz, 7.05 T, Bruker, Silberstreifen, Germany). Chemical shifts are given in ppm relative to TMS (^1^H, 0.0 ppm) and CDCl_3_ (^13^C, 77.0 ppm). Coupling constants are given in Hertz.
- UV-vis measurements: UV-vis absorption spectra were recorded on a UVICON XL spectrophotometer (Bio-Tex Instruments, Varanasi, India). All experiments were carried out at 298 K, using quartz cuvettes with an optical path of 1 cm.
- Steady-state fluorescence measurements: Fluorescence spectra were recorded at 298 K using an AMINCO Bowman Series 2 spectrofluorimeter (Aminco Bowman, Silver Spring, MD, USA) with 4.0-nm bandwidth for excitation and emission and quartz cuvettes with optical paths of 1 cm. The excitation wavelength was fixed at 280 nm.
- Time-resolved fluorescence measurements: Fluorescence decays were measured using the time-correlated single-photon-counting method on an FL-900 spectrofluorimeter from Edinburgh Analytical Instruments (Edinburgh, UK). The excitation source was a hydrogen nanosecond flash lamp with a repetition rate of 40 kHz, an excitation pulse width shorter than 1 ns, and a temporal resolution of 100 ps.
- Transmission electron microscopy: TEM images were obtained with a JEOL JEM 2100 transmission electron microscope (JEOL, Peabody, MA, USA) operating at an acceleration voltage of 200 kV.

### 3.2. Synthesis of Nonionic Ligands Ige_n_S-

**Scheme 2:**
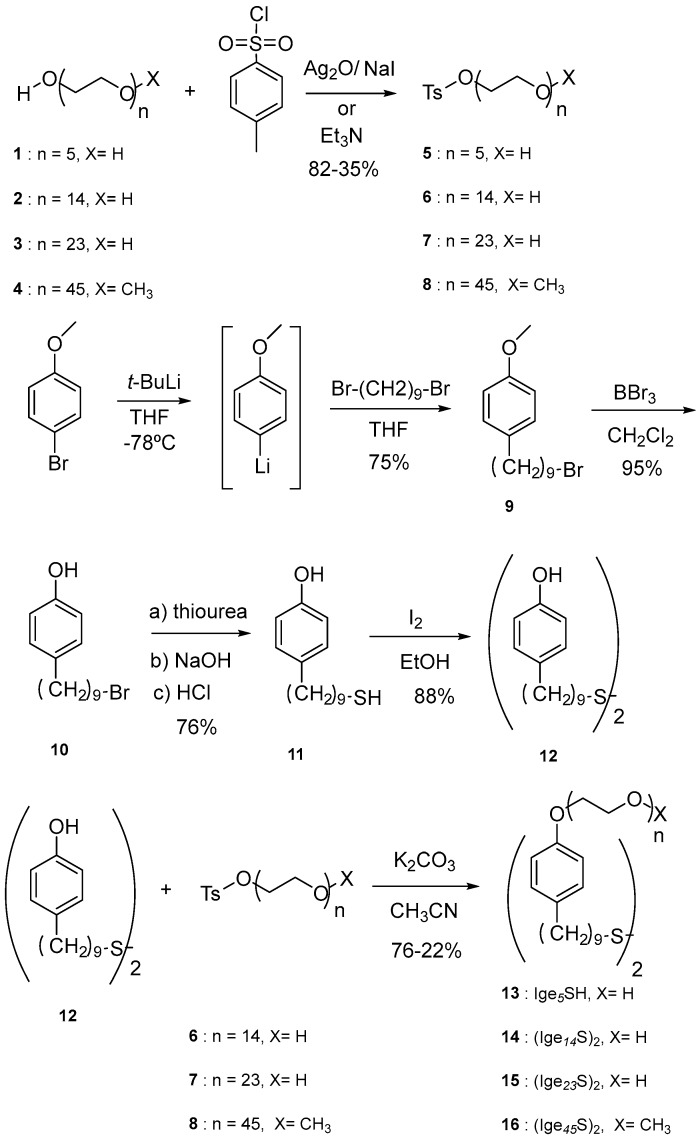
Synthesis of compounds **13**–**16**.

All starting materials and reagents were obtained from commercial sources and used without further purification. Anhydrous solvents were distilled under argon following standard procedures. All experiments were carried out under argon atmosphere using standard Schlenk techniques. Flash chromatography was performed over silica gel 60 (230–400 mesh). Ige_5_SH (**13**) (Scheme 2) was obtained from tosylate **5** according to a synthetic procedure described previously by us [31].

General procedure for the synthesis of tosylates **6** and **7** (Scheme 2) [56,57]: To a stirred solution of 1.6 mmol of **2** (or **3**) in 50 mL of CH_2_Cl_2_ at 0 °C were added 0.70 g (3 mmol) of Ag_2_O, 0.30 g (2 mmol) of NaI, and 0.34 g (1.8 mmol) of TsCl. The reaction mixture was stirred at room temperature for 3 days (the evolution of the reaction was followed by thin-layer chromatography, TLC). After filtration and evaporation of the solvent at reduced pressure, the residue was purified by flash chromatography (silica gel, CH_2_Cl_2_/MeOH 9:1).

**6:** Yield: 82%; ^1^H NMR (300 MHz, CDCl_3_, 25 °C, TMS): *δ* 7.82–7.74 (m, 2H, Ph), 7.37–7.30 (m, 2H, Ph), 4.14 (t, ^3^*J*(H,H) = 4.7 Hz, 2H, SO_2_-O-*CH*_2_-), 3.77–3.53 (m, 52H, O-*CH*_2_*-CH*_2_-O), 3.01 (brs, 1H, *OH*), 2.44 (s, 3H, Ph-*CH_3_*) ppm; ^13^C NMR (75 MHz, CDCl_3_, 25 °C, TMS): *δ* 144.4, 132.5, 129.4, 127.4, 72.1, 70.1, 70.0, 70.0, 69.7, 68.9, 68.1, 61.0, 21.1 ppm.

**7:** Yield: 80%; ^1^H NMR (300 MHz, CDCl_3_, 25 °C, TMS): *δ* 7.82–7.76 (m, 2H, Ph), 7.37–7.30 (m, 2H, Ph), 4.15 (t, ^3^*J*(H,H) = 4.7 Hz, 2H SO_2_-O-*CH_2_*-), 3.91–3.34 (m, 94H, O-*CH_2_-CH_2_*-O), 2.75 (brs, 1H, *OH*), 2.44 (s, 3H, Ph-*CH_3_*) ppm; ^13^C NMR (75 MHz, [D_6_]DMSO, 25 °C, TMS): *δ* 144.9, 133.2, 130.1, 127.6, 72.3, 70.0, 69.8, 69.7, 69.6, 67.9, 60.2, 54.8, 21.1 ppm.

Synthesis of tosylate **8** (Scheme 2) [58]: To a stirred solution of 1.0 mmol of **4** in 30 mL of CH_2_Cl_2_ at 0 °C were added 0.72 g (7.2 mmol) of Et_3_N and 0.95 g (5 mmol) of TsCl. The reaction mixture was stirred at room temperature for 2 days (the evolution of the reaction was followed by TLC). The tosylate was obtained by precipitation in cold Et_2_O and recrystallization from EtOH/Et_2_O.

**8:** Yield: 41%; ^1^H NMR (300 MHz, CDCl_3_, 25 °C, TMS): *δ* 7.82–7.77 (m, 2H, Ph), 7.37–7.31 (m, 2H, Ph), 4.15 (m, 2H SO_2_-O-*CH*_2_-), 3.90–3.38 (m, 182H, O-*CH*_2_*-CH*_2_-O), 3.37 (s, 3H, O-*CH*_3_), 2.44 (s, 3H, Ph-*CH*_3_) ppm.

Synthesis of compound **9** (Scheme 2): To a stirred solution of 1.38 g (7.38 mmol) of *p*-bromoanisole in 60 mL of THF at −78 °C under argon atmosphere were added 14.8 mmol of *t*-BuLi (1.7 M, 8.7 mL). After 5 min, a solution of 1,9-dibromononane (8.46 g, 29.6 mmol) in 50 mL of THF was added to the flask at −78 °C. After 2 days at 25 °C, water (100 mL) was added to the reaction mixture. After extraction with CH_2_Cl_2_ (3 × 30 mL), the organic solution was dried over MgSO_4_. Evaporation of the solvent under reduced pressure was followed by distillation of the excess of 1,9-dibromononane (119 °C, 0.33 mm Hg) and purification of the remaining residue by flash chromatography (silica gel, hexane), affording compound **9** (1.73 g, 75%) [59]. ^1^H NMR (300 MHz, CDCl_3_, 25 °C, TMS): *δ* 7.12–7.06 (m, 2H, Ph), 6.86–6.80 (m, 2H, Ph), 3.79 (s, 3H, O-*CH_3_*), 3.41 (t, ^3^*J*(H,H) = 6.8 Hz, 2H, -*CH*_2_-Br), 2.54 (t, ^3^*J*(H,H) = 7.4 Hz, 2H, Ph-*CH_2_*-), 1.85 (q, ^3^*J*(H,H) = 7.3 Hz, 2H, -*CH*_2_-), 1.58 (m, 2H, -*CH*_2_-), 1.42 (m, 2H, -*CH*_2_-), 1.30 (m, 8H, -*CH*_2_-) ppm; ^13^C NMR (75 MHz, CDCl_3_, 25 °C, TMS): *δ* 157.5, 134.7, 129.1, 113.5, 55.1, 34.9, 33.8, 32.8, 31.7, 29.3, 29.3, 29.1, 28.7, 28.1 ppm.

Synthesis of compound **10** (Scheme 2): To a stirred solution of 1.0 g (3.2 mmol) of **9** in 40 mL of CH_2_Cl_2_ at −10 °C under argon atmosphere was added 6.4 mmol of BBr_3_ (1 M, 6.4 mL). After 24 h at 25 °C, water (100 mL) was added and the resulting mixture was extracted with CH_2_Cl_2_ (2 × 15 mL). The organic solution was dried over MgSO_4_. Evaporation of the solvent under reduced pressure and purification of the residue by flash chromatography (silica gel, hexane/EtOAc 9:1) yielded 0.90 g (95%) of **10**. ^1^H NMR (300 MHz, CDCl_3_, 25 °C, TMS): δ 7.08–7.00 (m, 2H, Ph), 6.78–6.70 (m, 2H, Ph), 4.39 (bs, 1H, *OH*), 3.40 (t, ^3^*J*(H,H) = 7.0 Hz, 2H, -*CH*_2_-Br), 2.53 (t, ^3^*J*(H,H) = 7.2 Hz, 2H, Ph-*CH_2_*-), 1.85 (q, ^3^*J*(H,H) = 7.6 Hz, 2H, -*CH*_2_-), 1.56 (m, 2H, -*CH*_2_-), 1.41 (m, 2H, -*CH*_2_-), 1.29 (m, 8H, -*CH*_2_-) ppm; ^13^C NMR (75 MHz, CDCl_3_, 25 °C, TMS): *δ* 153.5, 135.3, 129.6, 115.2, 35.2, 34.2, 33.0, 31.8, 29.5, 29.5, 29.3, 28.9, 28.3 ppm; MS (70 eV): *m*/*z* (%): 300 (21) [*M^+^* + 2], 298 (22) [*M^+^*], 107 (100), 77 (16); HRMS (EI): calculated for C_15_H_23_OBr [*M^+^*]: 298.0926, found: 298.0926.

Synthesis of compound **11** (Scheme 2): To a stirred solution of 365 mg (1.22 mmol) of **10** in 25 mL of EtOH under argon atmosphere was added 467 mg (6.1 mmol) of thiourea. After refluxing for 24 h, 2 mL of 2 M NaOH was added and the reaction mixture was refluxed for additional 24 h. The flask was cooled at 0 °C and the solution was acidulated (pH = 2). After stirring at 25 °C for 24 h, the mixture was extracted with CH_2_Cl_2_ (3 × 20 mL) and the organic solution was dried over MgSO_4._ Evaporation of the solvent under reduced pressure and purification of the residue by flash chromatography (silica gel, hexane/EtOAc 9:1) yielded 235 mg (76%) of **11**. ^1^H NMR (300 MHz, CDCl_3_, 25 °C, TMS): *δ* 7.06–7.00 (m, 2H, Ph), 6.79–6.71 (m, 2H, Ph), 4.72 (s, 1H, *OH*), 2.58–2.46 (m, 4H, Ph-*CH*_2_- and –*CH*_2_-SH), 1.65–1.51 (m, 4H, -*CH*_2_-), 1.41–1.21 (m, 10H, -*CH*_2_-), 1.34 (t, ^3^*J*(H,H) = 7.7 Hz, 1H, *SH*) ppm; ^13^C NMR (75 MHz, CDCl_3_, 25 °C, TMS): *δ* 153.5, 135.3, 129.6, 115.2, 35.2, 34.1, 31.8, 29.5, 29.3, 29.1, 28.5, 24.8 ppm; MS (70 eV): *m*/*z* (%): 254 (2.6) [*M^+^* + 2], 253 (7.5) [*M^+^*+1], 252 (54) [*M^+^*], 107 (100); HRMS (EI): calculated for C_15_H_24_OS: 252.1542, found: 252.1540.

Synthesis of compound **12** (Scheme 2): To a stirred solution of 151 mg (0.6 mmol) of **11** in 5 mL of EtOH was added a solution of 176 mg (0.69 mmol) of I_2_ in 8 mL of EtOH. After 24 h at 25 °C, 30 mL of aqueous Na_2_S_2_O_3_ was added and the reaction mixture was extracted with CH_2_Cl_2_ (3 × 20 mL). The organic solution was dried over MgSO_4_ and, after evaporation of the solvent under reduced pressure and purification of the residue by flash chromatography (silica gel, hexane/EtOAc 9:1), **12** (132 mg, 88%) was obtained. ^1^H NMR (300 MHz, CDCl_3_, 25 °C, TMS): *δ* 7.07–7.00 (m, 4H, Ph), 6.78–6.70 (m, 4H, Ph), 4.72 (s, 2H, *OH*), 2.67 (t, ^3^*J*(H,H) = 7.3 Hz, 4H, -*CH_2_*-S-S), 2.52 (t, ^3^*J*(H,H) = 7.7 Hz, 4H, Ph-*CH_2_*-), 1.73–1.62 (m, 4H, -*CH*_2_-), 1.61–1.50 (m, 4H, -*CH*_2_-), 1.45–1.22 (m, 20H, -*CH*_2_-) ppm; ^13^C NMR (75 MHz, CDCl_3_, 25 °C, TMS): *δ* 153.5, 135.3, 129.6, 115.2, 39.3, 35.2, 31.8, 29.6, 29.6, 29.4, 29.3, 28.6 ppm. MS (70 eV): *m*/*z* (%): 504 (8) [*M^+^* + 2], 503 (19) [*M^+^*+1], 502 (66) [*M^+^*], 252 (14), 107 (100); HRMS (EI): calculated for C_30_H_46_O_2_S_2_: 502.2934, found: 502.2935.

General procedure for the synthesis of compounds **14**–**16** (Ige*_n_*S)_2_: To a stirred solution of 0.30 mmol of the corresponding tosylate **6**–**8** in 30 mL of acetonitrile under argon atmosphere were added 50 mg (0.1 mmol) of **12** and 60 mg (0.43 mmol) of K_2_CO_3_. After refluxing for 3 days, the reaction mixture was filtered and the solvent was evaporated under reduced pressure. Compounds **14** was purified by flash chromatography (silica gel, CH_2_Cl_2_/MeOH 9:1), while compounds **15** and **16** were recrystallized from EtOH/Et_2_O.

**14** (Ige_14_S)_2_: Yield: 76%; ^1^H NMR (300 MHz, CDCl_3_, 25 °C, TMS): *δ* 7.09–7.03 (m, 4H, Ph), 6.85–6.78 (m, 4H, Ph), 4.06 (t, ^3^*J*(H,H) = 4.6 Hz, 4H, Ph-O-*CH_2_*-), 3.80 (t, ^3^*J*(H,H) = 5.2 Hz, 4H, Ph-O-CH_2_-*CH*_2_-), 3.73–3.52 (m, 114H, O-*CH*_2_-*CH*_2_-O), 2.64 (t, ^3^*J*(H,H) = 7.3 Hz, 4H, -*CH_2_*-S), 2.49 (t, ^3^*J*(H,H) = 7.5 Hz, 4H, -*CH*_2_-Ph), 1.69–1.13 (m, 28H, -*CH*_2_-) ppm.

**15** (Ige_23_S)_2_: Yield: 71%; ^1^H NMR (300 MHz, CDCl_3_, 25 °C, TMS): *δ* 7.09–7.03 (m, 4H, Ph), 6.85–6.78 (m, 4H, Ph), 4.09 (t, ^3^*J*(H,H) = 4.6 Hz, 4H, Ph-O-*CH*_2_-), 3.83 (t, ^3^*J*(H,H) = 5.2 Hz, 4H, Ph-O-CH_2_-*CH_2_*-), 3.73–3.52 (m, 184H, O-*CH*_2_-*CH*_2_-O), 2.64 (t, ^3^*J*(H,H) = 7.3 Hz, 4H, -*CH*_2_-S), 2.51 (t, ^3^*J*(H,H) = 7.5 Hz, 4H, -*CH*_2_-Ph), 1.69–1.13 (m, 28H, -*CH*_2_-) ppm.

**16** (Ige_45_S)_2_: Yield: 38%; ^1^H NMR (300 MHz, CDCl_3_, 25 °C, TMS): *δ* 7.09–7.03 (m, 4H, Ph), 6.84–6.79 (m, 4H, Ph), 4.10 (t, ^3^*J*(H,H) = 4.6 Hz, 4H, Ph-O-*CH*_2_-), 3.87–3.47 (m, 356H, O-*CH*_2_-*CH*_2_-O), 3.38 (s, 6H, O-*CH*_3_), 2.67 (t, ^3^*J*(H,H) = 7.3 Hz, 4H, -*CH*_2_-S), 2.53 (t, ^3^*J*(H,H) = 7.5 Hz, 4H, -*CH*_2_-Ph), 1.67–1.20 (m, 28H, -*CH*_2_-) ppm.

### 3.3. Synthesis of Spherical Gold Nanoparticles

Materials and methods: All the starting materials were obtained from commercial suppliers and used without further purification. The gold(III) chloride (HAuCl_4_·H_2_O, 99.999%), sodium citrate (≥99%), sodium borohydride (NaBH_4_, 99.99%), L-ascorbic acid (≥99%), cetyltrimethylammonium chloride solution (CTAC, 25 wt % in H_2_O), cetyltrimethylammonium bromide (CTAB, ≥99%), and poly(ethylene glycol) methyl ether thiol (PEG*_n_*SH, av. *M*w = 6000) used for the synthesis and coating of AuNPs were purchased from Sigma-Aldrich (Madrid, Spain).

Synthesis of citrate-stabilized AuNPs: In a typical synthesis [45], a solution of HAuCl_4_ (0.4 mM, 50 mL) was heated to boiling. A solution of sodium citrate (1 wt %, 2 mL) was then quickly injected into the above solution under vigorous stirring. The mixture was kept boiling for 10 min until the color changed to red. Then, at room temperature, the mixture was centrifuged (6500 rpm, 90 min) to remove the excess of sodium citrate and the precipitate was redispersed in the same volume of water. The resulting citrate-stabilized gold nanospheres (cit-AuNPs) presented a diameter of 15 ± 2 nm, as determined from TEM images (Figure 1a).

Synthesis of CTAC-stabilized AuNPs: AuNPs were synthesized through a successive seed-mediated growth process [60]. First, nanometric Au clusters were prepared by chemical reduction of HAuCl_4_ using NaBH_4_ as the reducing agent and CTAB as the stabilizing agent. In brief, a freshly prepared aqueous solution of NaBH_4_ (600 µL, 10 mM) was quickly added to 10 mL of an aqueous solution of CTAB (100 mM) and HAuCl_4_ (0.25 mM), resulting in a brownish solution. Then, for the synthesis of AuNPs of ca. 10 nm of average diameter, 2 mL of an aqueous solution of HAuCl_4_ (0.5 mM) was rapidly added to 22 mL of an aqueous solution of CTAC (18.8 mM) containing 1.5 mL of ascorbic acid (100 mM) and 50 µL of the freshly prepared cluster solution under magnetic stirring, resulting in a yellowish mixture. The solution color gradually turned from yellowish to red. The product was centrifuged twice at 10,000 rpm for 1 h and redispersed in 1 mL of an aqueous CTAC solution (20 mM). Finally, to increase the size of the previously synthesized AuNPs to 55 ± 5 nm (Figure 6a), an aqueous HAuCl_4_ solution (40 mL, 5 mM) was slowly injected (20 mL/h) into an aqueous CTAC solution (200 mM) containing 3.24 mL of an ascorbic acid solution (100 mM) and 150 µL of the as-prepared AuNP colloid (ca. 10 nM). During HAuCl_4_ solution injection, the color of the colloid solution gradually turned from red to purple. The injection process was stopped when the solution color changed suddenly to deep translucent red. The product was centrifuged twice at 3000 rpm for 15 min and redispersed in 5 mL of an aqueous CTAC solution (25 mM). The whole synthesis process was performed at room temperature.

Ige*_n_*S- and PEG*_n_*SH ligand replacement: An aqueous solution of the dimeric precursor (Ige*_n_*S)_2_ (6 mM, 5 mL) previously sonicated for 5 min was added to an AuNP solution stabilized with citrate (1 nM, 25 mL) under stirring for 3 h. Then, the excess of free ligand was removed from the solution by four centrifugation cycles (6500 rpm, 90 min). In each cycle, the supernatant was removed and the precipitate was redispersed in the same volume of water (Figure 1b). No significant differences in the size of Ige*_n_*S-AuNPs were observed with respect to the initial AuNPs by TEM. For a 1 nM AuNP solution, the final concentration of capping surfactant at the surface of the nanocrystal was estimated to be ca. 1 µM from the UV-vis spectrum of the supernatants, employing the molar absorptivity of the Igepal surfactant in water [31]. The same experimental procedure was employed for the coating of citrate- and CTAC-stabilized AuNPs with PEG*_n_*SH.

### 3.4. Calculations

The T-matrix method was initially developed by Waterman [61] and later improved by Mishchenko and others [50,51]. It is based on expanding the incident and scattered waves in appropriate vector spherical wave functions and relating these expansions by means of a transition (or T) matrix. This approach has proven to be extremely powerful and has been used, among others, to simulate electromagnetic, acoustic, and elastodynamic wave scattering by single and aggregated scatterers [62]. The code used in this manuscript (MSTM v3.0) was developed by Mackowski and Mishchenko [50,51] and simulates the electromagnetic scattering and absorption properties of an ensemble of spheres. The code is designed to run on either serial platforms or distributed-memory computer clusters. The large 2D array was simulated by calculating the near-field for an array with 15 × 15 particles and discarding the results at the borders to avoid spurious effects at the edges.

## Figures and Tables

**Table 1:** UV-vis spectral and photophysical properties of the monomeric ligand–gold nanoparticles (Ige*_n_*S-AuNPs) and cyclodextrin–monomeric ligand–gold nanoparticles (CD-Ige*_n_*S-AuNPs).

AuNP ^1^	*λ*_Ige*n*S-_^2^/nm	*λ*_LSPR_^3^/nm	*λ*_em_^4^/nm	*τ*_Ige*n*S-_^5^/ns	*τ*_α-CD_^6^/ns	*K*_b_^7^ × 10^−4^/M^−1^	*a* ^8^
Ige_5_S-	280	524	300	4.0 ± 0.1	6.4 ± 0.1	3.7 ± 0.4	1.52 ± 0.01
Ige_14_S-	280	524	300	4.3 ± 0.1	6.2 ± 0.1	1.7 ± 0.1	1.53 ± 0.01
Ige_23_S-	280	524	300	4.4 ± 0.1	6.3 ± 0.1	1.6 ± 0.2	1.50 ± 0.01
Ige_45_S-	280	524	300	4.5 ± 0.1	6.7 ± 0.1	1.3 ± 0.2	1.48 ± 0.02

^1^ All the samples were recorded at 298 K in water (1 nM of AuNPs). ^2^ Maximum of the UV-vis absorption band of the ligand in Ige*_n_*S-AuNPs (3 mM of Ige*_n_*S-). ^3^ Maximum of the localized surface plasmon resonance (LSPR) band of Ige*_n_*S-AuNPs (~1 µMµ of Ige*_n_*S-). ^4^ Maximum of the emission spectrum of Ige*_n_*S-AuNPs (~1 µM of Ige*_n_*S-; *λ*_exc_ = 280 nm). ^5^ Fluorescence decay time of Ige*_n_*S-AuNPs (~1 µM of Ige*_n_*S-; *λ*_exc_ = 280 nm). ^6^ Fluorescence decay time of CD-Ige*_n_*S-AuNPs (~1 µM of Ige*_n_*S- and 1 mM of α-CD; *λ*_exc_ = 280 nm). ^7^ Apparent association constant between the aromatic moiety of the ligand and α-CD obtained from the emission spectra of CD-Ige*_n_*S-AuNPs at different α-cyclodextrin (α-CD) concentrations (~1 µM of Ige*_n_*S- and 1–100 µM of α-CD). ^8^ Estimated ratio of the quantum yields and absorptivity of the complexed and free state of the aromatic ring (*a = ε*_CD-Ige_*_n_*_S-AuNP_ *ϕ*_CD-Ige_*_n_*_S-AuNP_*/ε*_Ige_*_n_*_S-AuNP_ *ϕ*_Ige_*_n_*_S-AuNP_).

**Table 2:** Interparticle distance in the arrays according to the number of oxyethylene units in CD-Ige*_n_*S-AuNPs.

AuNPs ^1^	*n*_OE_^2^	*n*_*α*-CD_^3^	*d*_th_^4^	*d*_exp_^5^
Ige_5_S-	5	5	4.0 ± 0.5	4.1 ± 0.5
Ige_14_S-	14	9	7.1 ± 0.5	7.8 ± 0.5
Ige_23_S-	23	14	11.1 ± 0.5	10.9 ± 0.5
Ige_45_S-	45	25	19.8 ± 0.5	20.0 ± 0.5

^1^ All the samples were prepared by drop-casting CD-Ige*_n_*S-AuNPs (~1 µM of Ige*_n_*S-, 1 nM of AuNPs, and 1 mM α-CD). ^2^ Number of oxyethylene units of the corresponding ligand. ^3^ Estimated number of macrocycles of polyrotaxanes at the surface of AuNPs. ^4^ Theoretical interparticle distance obtained from the estimated number of polyrotaxane macrocycles. ^5^ Interparticle distance obtained from the FFT analysis of the TEM micrographs.

## Supplementary Materials

The following are available online at http://www.mdpi.com/2079-4991/8/3/168/s1, Figure S1: ^1^H NMR spectrum of compound **10**, Figure S2: ^13^C NMR spectrum of compound **10**, Figure S3: ^1^H NMR spectrum of compound **11**, Figure S4: ^13^C NMR spectrum of compound **11**, Figure S5: ^1^H NMR spectrum of compound **12**, Figure S6: ^13^C NMR spectrum of compound **12**, Figure S7: ^1^H NMR spectrum of compound **14**, Figure S8: ^1^H NMR spectrum of compound **15**, Figure S9: ^1^H NMR spectrum of compound **16**.
