# Supplementary material for: Supramolecular Control over the Interparticle Distance in Gold Nanoparticle Arrays by Cyclodextrin Polyrotaxanes
Source: Nanomaterials (Basel). 2018 Mar 16;8(3):168. doi: 10.3390/nano8030168 (PMC5869659; doi:10.3390/nano8030168)
Supplement: Supplementary file 1 [file nanomaterials-08-00168-s001.pdf]

Supplementary Materials

Supramolecular Control over the Interparticle Distance in Gold Nanoparticle Arrays by Cyclodextrin Polyrotaxanes

Joao Paulo Coelho [1], José Osío Barcina [2], Elena Junquera [1], Emilio Aicart [1], Gloria Tardajos [1], Sergio Gómez-Graña [3], Pablo Cruz-Gil [2], Cástor Salgado [2], Pablo Díaz-Núñez [4], Ovidio Peña-Rodríguez [4] and Andrés Guerrero-Martínez [1,*]

[1] Departamento de Química Física, Universidad Complutense de Madrid, Avenida Complutense s/n, 28040 Madrid, Spain; jpaulogaio@gmail.com (J.P.C.); junquera@quim.ucm.es (E.J.); aicart@quim.ucm.es (E.A.); tardajos@quim.ucm.es (G.T.)
[2] Departamento de Química Orgánica, Universidad Complutense de Madrid, Avenida Complutense s/n, 28040 Madrid, Spain; josio@quim.ucm.es
[3] Departamento de Química en Ciencias Farmacéuticas, Universidad Complutense de Madrid, Plaza Ramón y Cajal s/n, 28040 Madrid, Spain; segome02@ucm.es
[4] Instituto de Fusión Nuclear, Universidad Politécnica de Madrid, José Gutiérrez Abascal 2, 28006 Madrid, Spain; pablodn87@gmail.com (P.D.-N.); ovidio.pena@upm.es (O.P.-R.)
* Correspondence: aguerrero@quim.ucm.es; Tel.: +34-934-4274

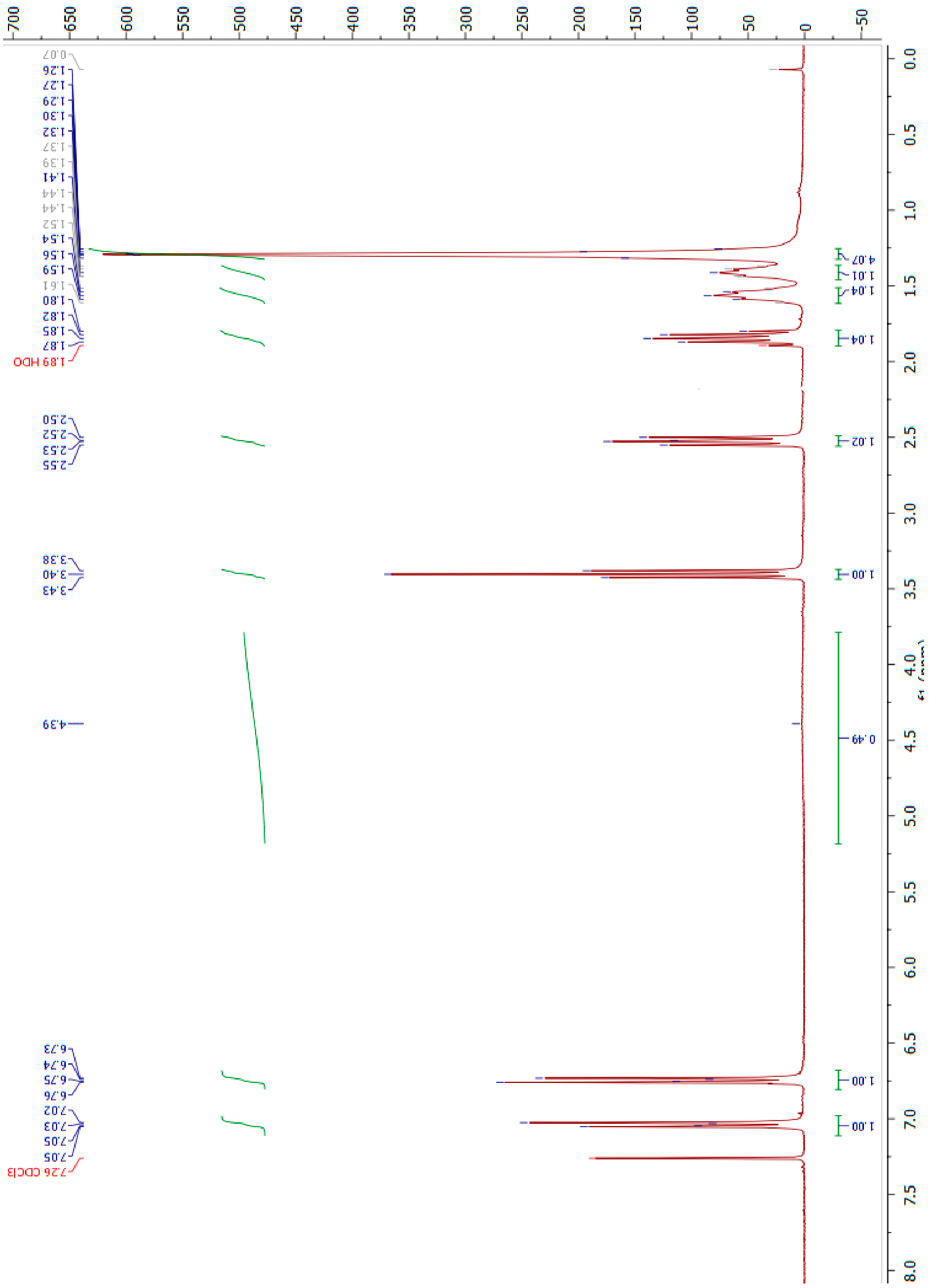

Figure S1. ^{1}H NMR spectrum of compound **10**.

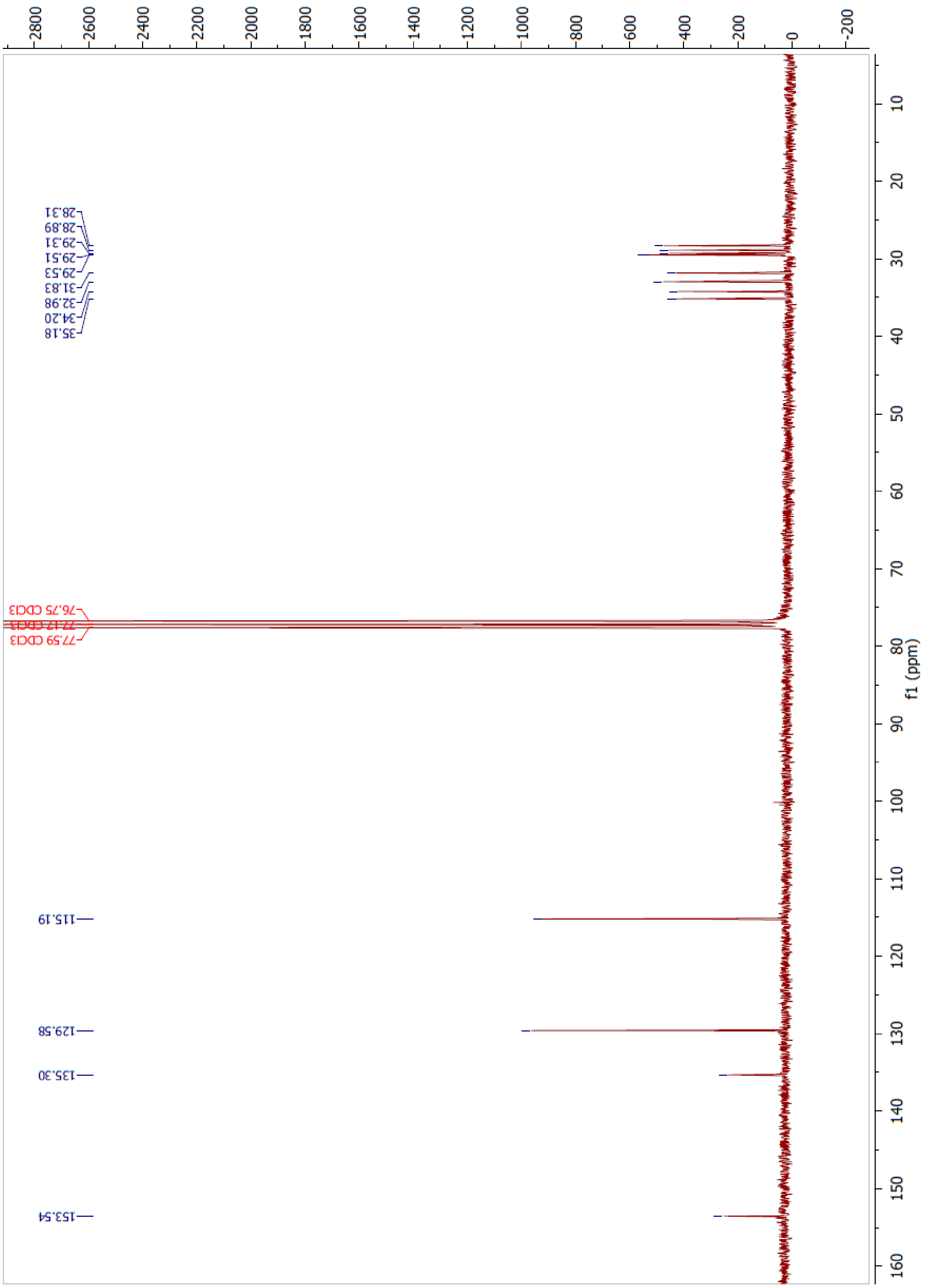

Figure S2. ^{13}C NMR spectrum of compound **10**.

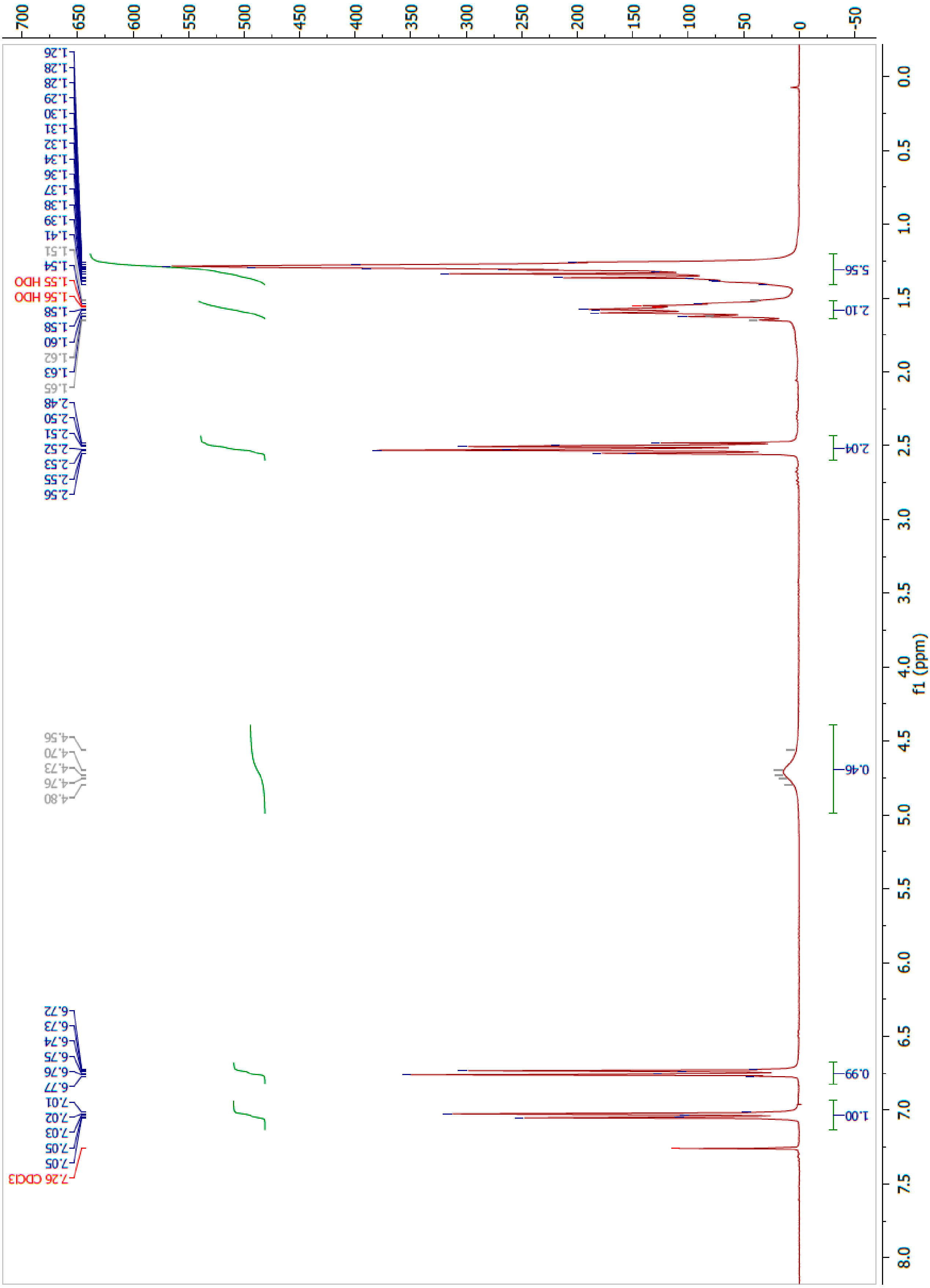

Figure S3. ^{1}H NMR spectrum of compound **11**.

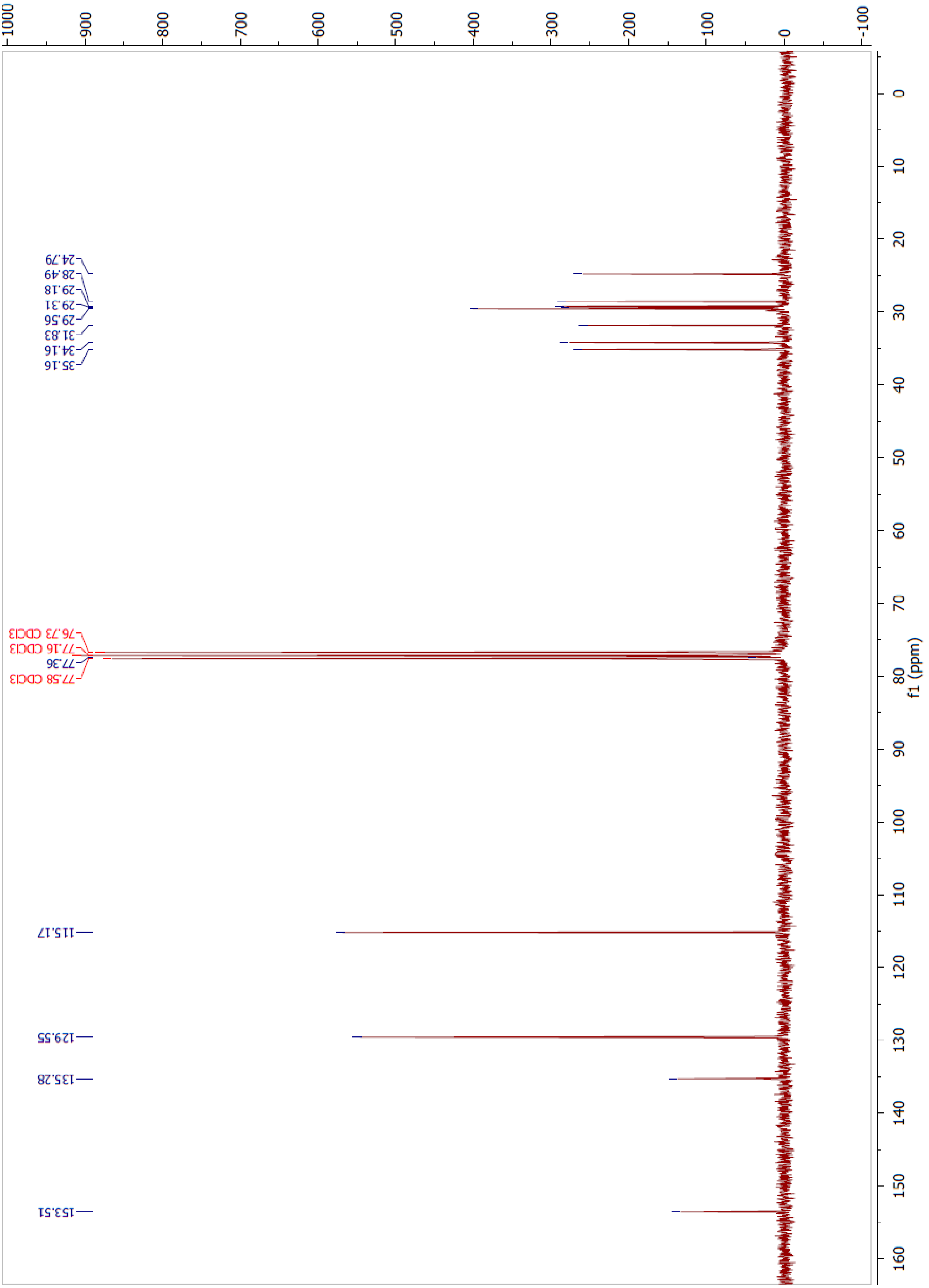

Figure S4. ^{13}C NMR spectrum of compound **11**.

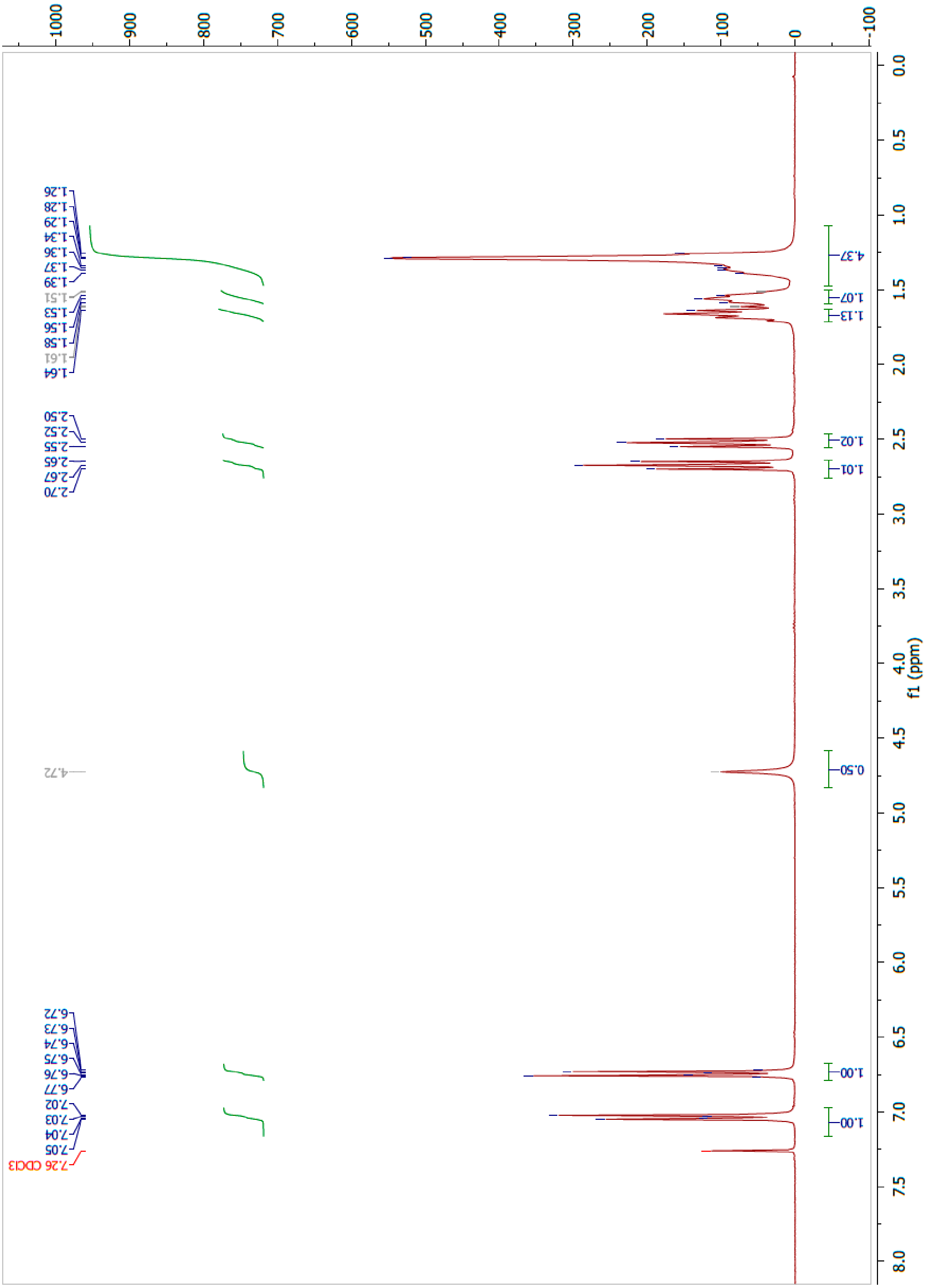

Figure S5. ^{1}H NMR spectrum of compound **12**.

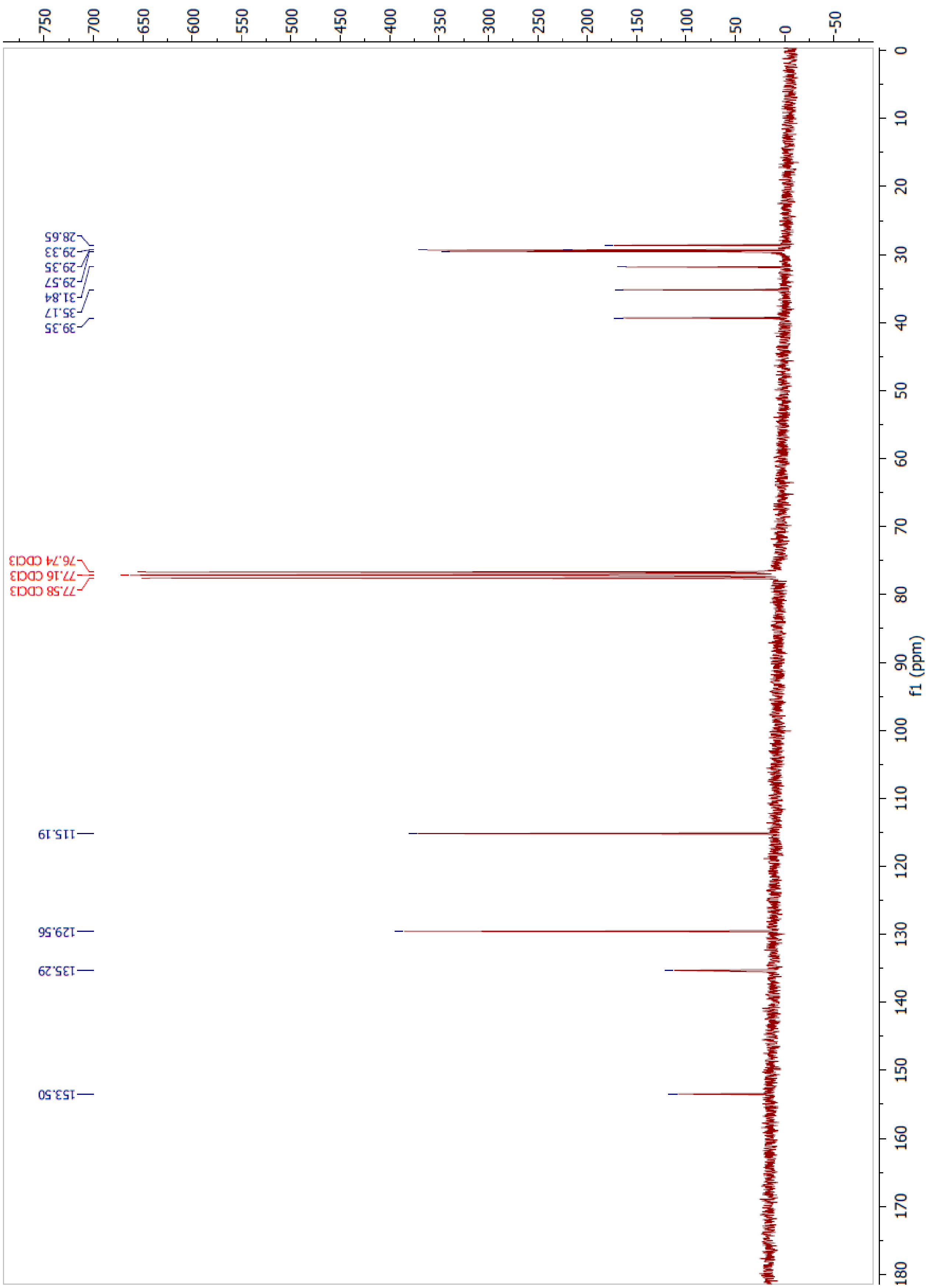

Figure S6. ^{13}C NMR spectrum of compound **12**.

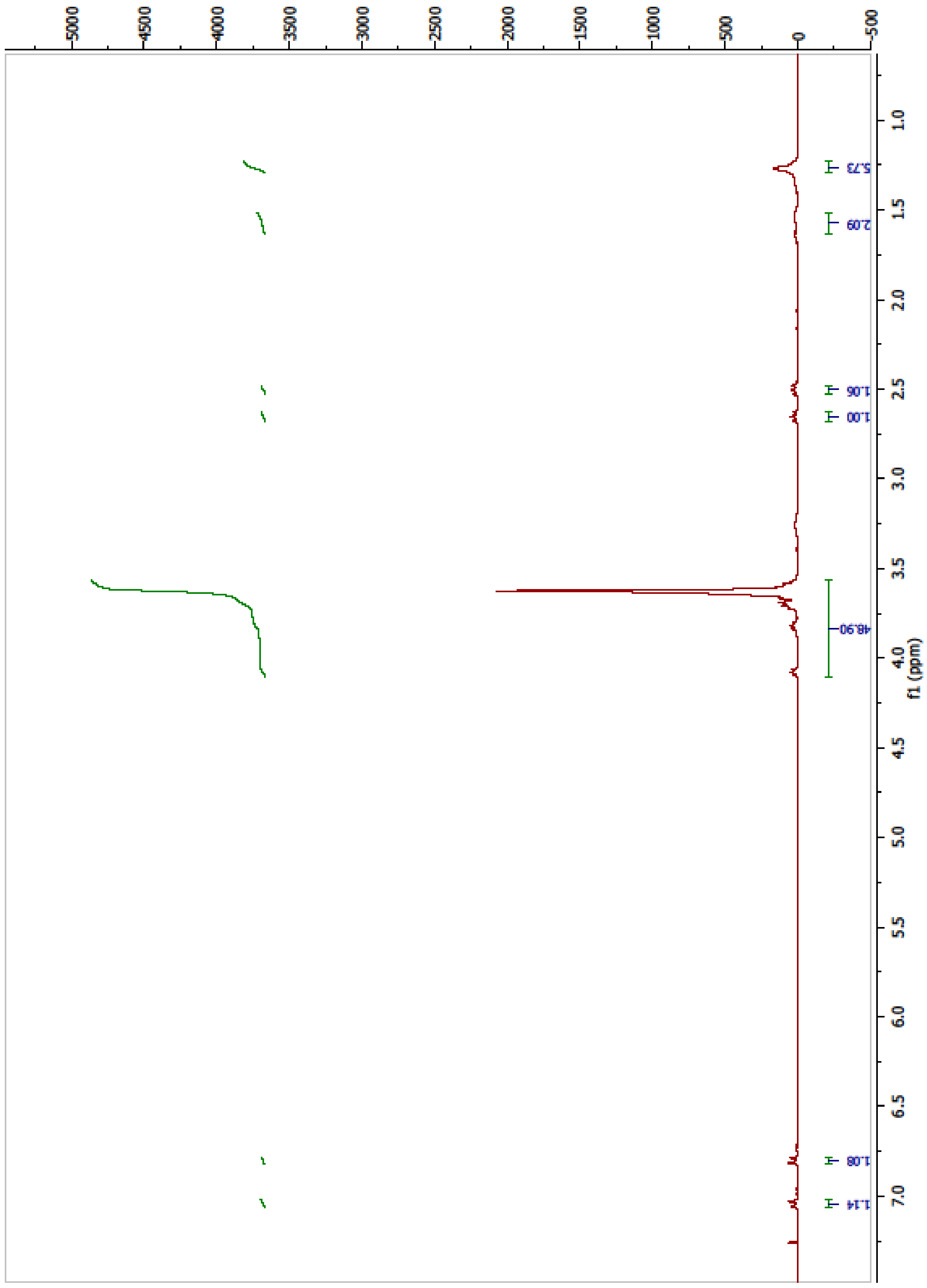

Figure S7. ^{1}H NMR spectrum of compound **14**.

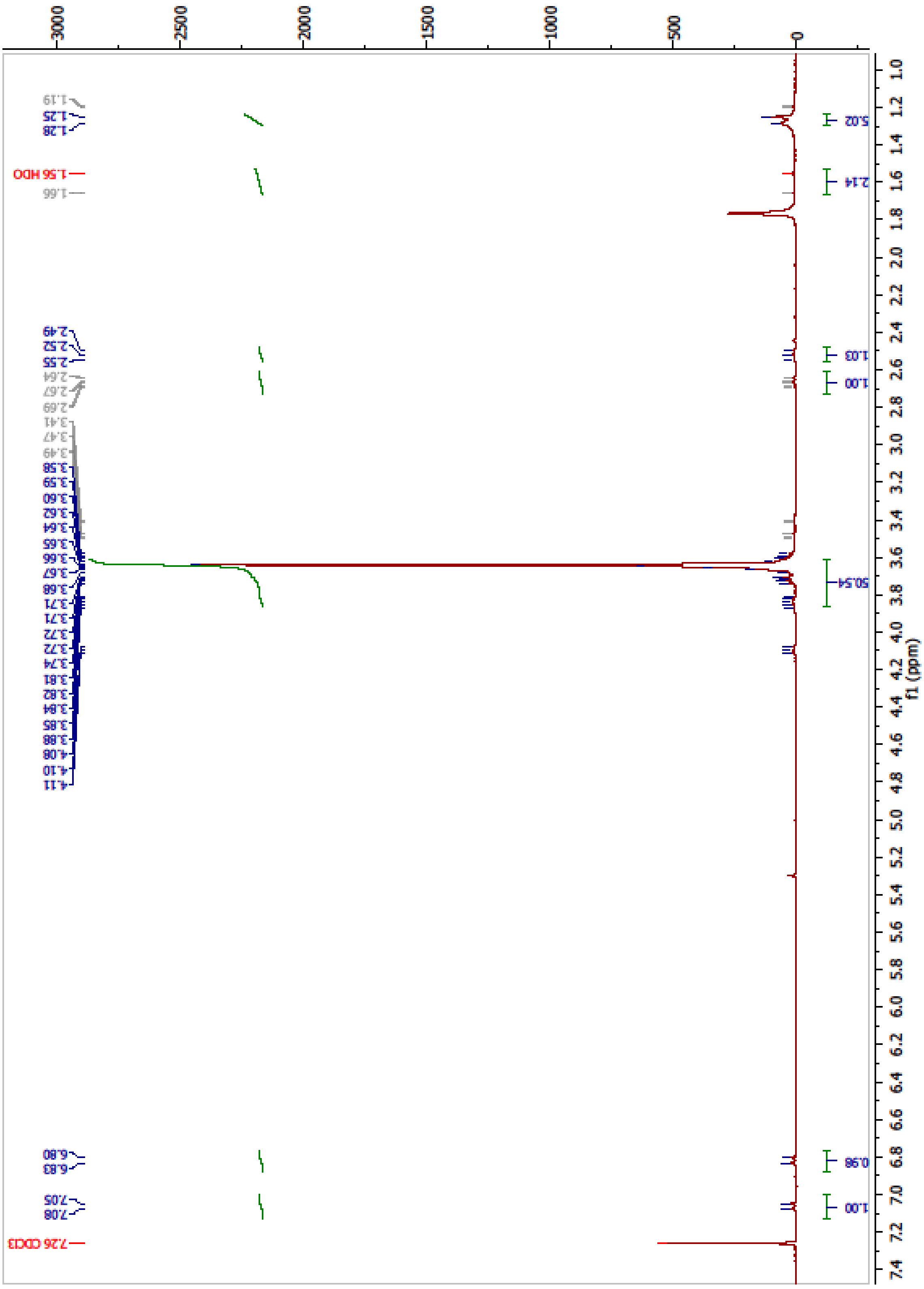

Figure S8. ^{1}H NMR spectrum of compound **15**.

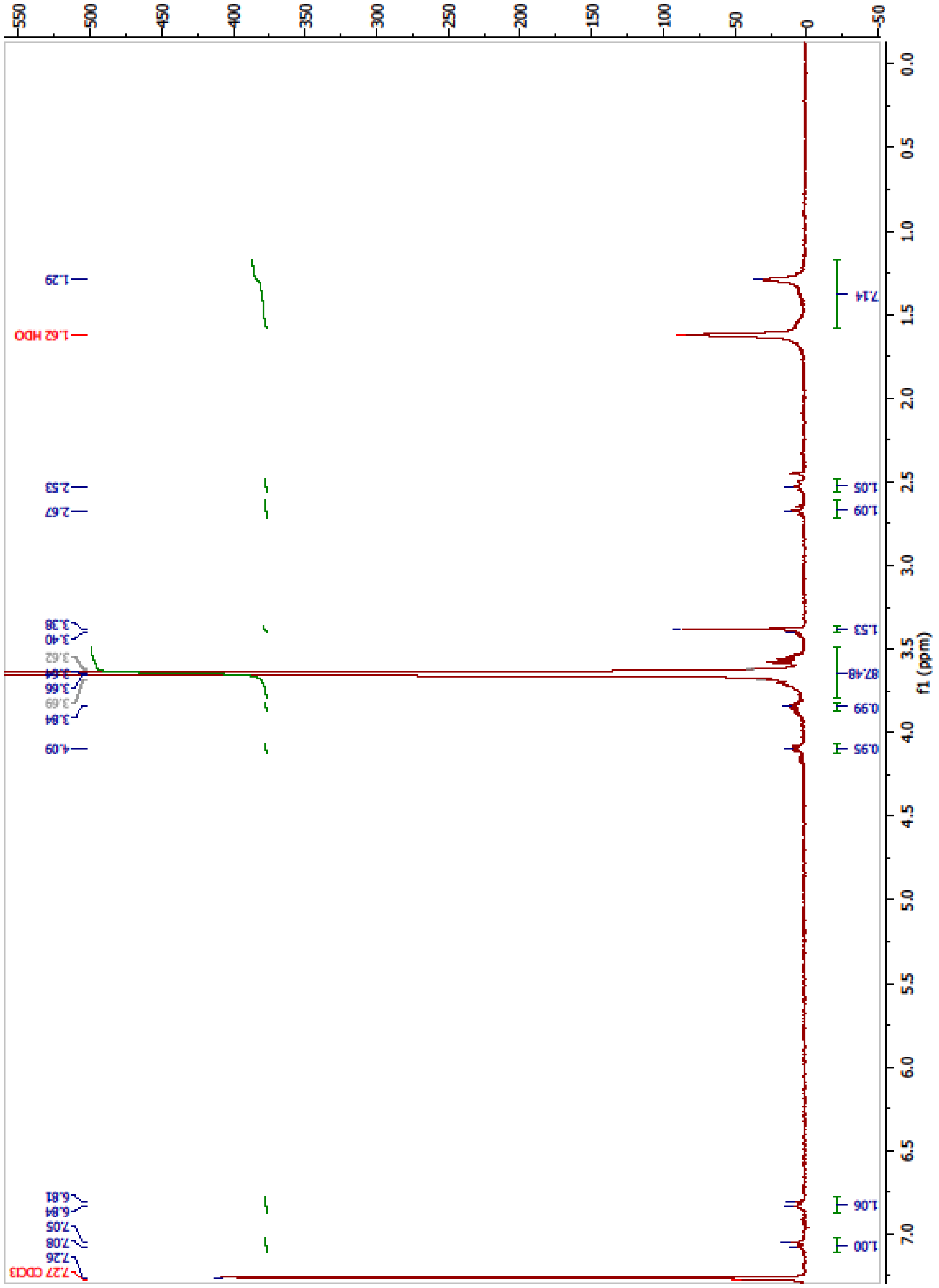

Figure S9. ^{1}H NMR spectrum of compound **16**.
